# O.5.3-6 Community-based physical activity promotion in Germany: a systematic overview and good practice projects

**DOI:** 10.1093/eurpub/ckad133.257

**Published:** 2023-09-11

**Authors:** Lea Dippon, Natalie Helsper, Simone Kohler, Philipp Weber, Leonie Birkholz, Alfred Rütten, Klaus Pfeifer, Jana Semrau

**Affiliations:** Friedrich-Alexander-Universität Erlangen-Nürnberg; lea.j.dippon@fau.de; Friedrich-Alexander-Universität Erlangen-Nürnberg; lea.j.dippon@fau.de; Friedrich-Alexander-Universität Erlangen-Nürnberg; lea.j.dippon@fau.de; Friedrich-Alexander-Universität Erlangen-Nürnberg; lea.j.dippon@fau.de; Friedrich-Alexander-Universität Erlangen-Nürnberg; lea.j.dippon@fau.de; Friedrich-Alexander-Universität Erlangen-Nürnberg; lea.j.dippon@fau.de; Friedrich-Alexander-Universität Erlangen-Nürnberg; lea.j.dippon@fau.de; Friedrich-Alexander-Universität Erlangen-Nürnberg; lea.j.dippon@fau.de

## Abstract

**Purpose:**

Approaches to community-based physical activity promotion (c-PAP) are recommended to counteract physical inactivity in the population. The aim of this study was to present a systematic overview of the practice of c-PAP in Germany. Furthermore, we aimed to identify good practice projects to provide guidance for the implementation and scale up of c-PAP.

**Methods:**

In the first step, we identified projects by searching 4 scientific databases and 21 project databases. For each project, we extracted data on “federal state”, “consideration of health equity” and “implementation in urban or rural areas”. In addition, we sorted the included projects into different types of community-based approaches. In the next step, we assessed projects with a documented process and/or outcome evaluation using quality criteria for the phases of conceptualization, implementation, and evaluation. Projects that fulfilled at least 50% of the quality criteria were selected as good practice projects.

**Results:**

We identified a total of 240 c-PAP projects. Rural areas primarily implemented environmental approaches while urban areas focused more on multi-component approaches as well as offers and events. 45 projects showed adequate documentation of the process and/or outcome evaluation, of which 17 projects were identified as good practice.

**Conclusions:**

Overall, there is potential for improvement in addressing health equity, an active participation of people with social disadvantages over the entire project cycle as well as the implementation of multi-component approaches. This requires funding programs that allow for good practice projects, which establish structures and particularly reach disadvantaged communities. A jointly adopted standard by stakeholders from policy, practice, and research for the application of quality criteria along with a classification for good practice could be an asset for further strategic development of c-PAP in Germany.

**Funding Source:**

This work was supported by the Federal Centre of Health Education (BZgA) on behalf of and with funds from the statutory health insurances according to § 20a SGB V in the context of the GKV Alliance for Health (www.gkv-buendnis.de).

